# The Impact of Dog-Assisted Therapy Among Children and Adolescents with Autism Spectrum Disorder: A Systematic Review

**DOI:** 10.3390/children11121499

**Published:** 2024-12-09

**Authors:** Paula Galvany-López, Manuel Martí-Vilar, Sergio Hidalgo-Fuentes, Javier Cabedo-Peris

**Affiliations:** Basic Psychology Department, Faculty of Psychology and Speech Therapy, Universitat de València, 46010 Valencia, Spain; paugalo@alumni.uv.es (P.G.-L.); sergio.hidalgo@uv.es (S.H.-F.); jacape2@alumni.uv.es (J.C.-P.)

**Keywords:** Autism Spectrum Disorder, dog-assisted therapy, children, adolescence, therapeutic intervention

## Abstract

Background and Objectives: Animal-assisted therapies have been utilized in various profiles to improve people’s quality of life. This systematic review aims to assess the impact of dog-assisted therapies (DAT) on children and adolescents with Autism Spectrum Disorder (ASD). The benefits provided, the feasibility of implementation, and potential limitations are analysed. Methods: An exhaustive search was carried out in the following databases: Web of Science (WoS), Scopus, ERIC, PubMed, PubPsych, CINAHL, PsycInfo and Dialnet. The procedure was preregistered on PROSPERO. Results: This review included 19 scientific articles. Of these, 84% suggest that DAT has a positive impact on communication and social interaction during the development of children and adolescents diagnosed with ASD. The dog is often perceived as a communication facilitator that serves as a reinforcer, capturing the individual’s attention. Emotional benefits have also been reported, including reducing stereotyped and self-harming behaviours. Additionally, an increase in smiling frequency and self-esteem levels was observed. Conclusions: These findings highlight the potential of DAT as a component of socio-educational intervention for children and adolescents diagnosed with ASD. However, further research is necessary due to the limited sample sizes in the studies reviewed.

## 1. Introduction

### 1.1. Autism Spectrum Disorder (ASD)

The recent DSM-5-TR edition, as outlined by the APA [1], ASD is within the category of neurodevelopmental disorders. To receive a diagnosis, an individual must exhibit persistent deficits in communication and social interaction alongside restrictive, repetitive, and stereotyped patterns of behaviors, activities, and interests. These impairments must manifest in early childhood and cause clinically significant disruptions in social, occupational, or other important areas of functioning. The manual defines three severity levels based on the degree of assistance required and the observable symptoms: Level 1, “Requiring support”; Level 2, “Requiring substantial support”; and Level 3, “Requiring very substantial support”. Additionally, it highlights the high rate of co-occurrence among neurodevelopmental disorders, meaning ASD is often associated with intellectual disabilities or attention-deficit/hyperactivity disorder (ADHD). Furthermore, language impairment may also accompany ASD, among other conditions.

It is worth noting that individuals previously diagnosed with Asperger’s disorder are now classified under ASD without intellectual or language impairment. Consequently, as this study adheres to the updated definition of ASD, Asperger’s disorder is included [1]. According to Castillo Bautista and Sánchez-Suricalday [2], the term ‘high-functioning autism’ refers to individuals with ASD who exhibit normative intelligence but face social and emotional challenges.

Studies indicate that the World Health Organization [3] estimates 1 in 100 children receive an autism diagnosis, an average figure considering variability across studies. However, there is disagreement about the causes of the substantial increase. Some suggest this rise may be attributed to methodological factors or changes in diagnostic criteria. Others propose it could reflect greater awareness of the issue or an actual increase in prevalence [4,5,6]. Notably, ASD is diagnosed three to four times more frequently in males than females, although research is being conducted to determine if this discrepancy is due to the evaluation techniques used [7,8]. The etiology of ASD remains unknown, but studies suggest it is multifactorial with a strong genetic influence [9].

Early detection and early stage intervention are critical for a positive prognosis. Given the primary needs of individuals with autism, interventions typically target the most affected areas. Examples of these are imitation, communication, and language, social initiation and motivation, and the development of motor and cognitive skills, among others. Emphasis is placed on encouraging spontaneous interaction initiated by the child [10].

According to Begeer et al. [11], the Theory of Mind (ToM)—the ability to infer others’ feelings and emotions—is a key prerequisite for effective social interactions. It is usually developed between the ages of 6 and 8. Impairments in ToM are recognized as a characteristic symptom of ASD and are addressed in therapeutic and educational interventions. Among other challenges faced by individuals with ASD, Paula-Pérez and Artigas-Pallarés [12] argue that high anxiety levels are associated with increased repetitive and stereotyped movements, object use, and speech, as well as an insistence on sameness. These behaviors serve as stress-relief and self-regulation mechanisms. Therefore, individuals with ASD learn best in predictable social environments with minimal ambiguity. Similarly, Calderón [13] underscores the importance of working on eye contact, gestural communication, and attentiveness to conversation partners.

### 1.2. Dog-Assisted Therapy (DAT)

Individuals with autism have access to various therapies [14,15,16], including complementary or alternative treatments [17]. Non-pharmacological therapies such as music therapy, hydrotherapy, and animal-assisted interventions (AAI) are examples of complementary approaches. According to the International Association of Human-Animal Interaction Organizations) [18], AAI encompasses structured interventions guided by specific goals designed to achieve therapeutic benefits in health, education, and social domains. This framework goes beyond the mere presence or exposure to animals, incorporating structured sessions with defined objectives.

AAI are becoming increasingly popular across various healthcare settings worldwide [19]. These programs are employed to support patients with a wide range of conditions, including mental health disorders and chronic illnesses such as cancer. The growing interest in AAI is driven by evidence suggesting that these interventions can enhance psychological well-being, alleviate stress, and address emotional and social needs, making them a valuable complement to traditional therapeutic approaches [20,21,22,23]. Other authors, such as Urinovsky and Cafiero [17], express skepticism about alternative therapies. They noted that they may offer potential benefits. However, the existing studies often lack rigor, are biased, or their quality ranges from low to moderate.

Among the various forms of Animal-Assisted Interventions (AAI), Dog-Assisted Therapy (DAT) stands out as a specific approach in which certified therapy dogs actively contribute to achieving therapeutic or educational goals. DAT is a structured and goal-directed therapy, implemented by healthcare professionals, that purposefully includes dogs in the therapeutic process to achieve specific therapeutic outcomes and improve health and well-being [24]. It involves active participation from individuals, focusing on achieving defined therapeutic goals, with outcomes systematically recorded. DAT differs from other animal-based practices, such as general AAI or service dog programs, which address different needs. Therapy dogs are integrated directly into structured therapeutic sessions, where they act as co-therapists, transitional objects, and social mediators, providing multisensory stimulation [25]. In contrast, service dogs are trained to assist with the daily needs of individuals with autism and their families, typically in non-therapeutic settings like the home. It is important to note that DAT, as a form of AAI, serves as a tool to facilitate and motivate interventions, breaking down barriers and enhancing engagement. However, it is not intended to replace conventional therapies [26].

The outcomes of DAT are categorized into three main areas [27]: (1) improvements in social skills, such as empathy and increased verbal communication, (2) psychological effects, including enhanced concentration, self-esteem, and motivation, and (3) neurobiological effects, such as reductions in heart rate, blood pressure, and cortisol levels.

### 1.3. The Current Study

Despite the different roles that dogs play in interacting with people with autism, the systematic review will focus exclusively on the use of therapy dogs with children and adolescents with ASD. This choice is due to the interest in specifically understanding the effectiveness of DAT for a school-age population.

This work acknowledges the existence of a previous review on the same topic [28], which focused exclusively on children, whereas ours expands the scope to include both children and adolescents. Additionally, it incorporates four extra databases and updates the information to cover data up to 2023, encompassing the entire year. This review also delves deeper into critical aspects, such as the instruments used and their reliability, the study design, and the inclusion of a control group. Furthermore, it places particular emphasis on factors that may influence the therapies, including the duration of the intervention and the qualifications of the individuals conducting them.

The aim of this study is exclusively focused on DAT with children and adolescents diagnosed with ASD. It seeks to evaluate the impact and benefits of these therapies while exploring factors influencing their efficacy and feasibility. The research question (RQ) was formulated using the PICO (Patients or Problem, Intervention, Comparison and Outcomes) methodology. Where (P) is children and adolescents with autism spectrum disorder, (I) is dog-assisted therapy, (C) is the presence of a control group, and (O) is the impact, improvements, and potential benefits.

RQ: *What are the results of applying DAT in children and adolescents diagnosed with ASD compared to a control group?*

## 2. Materials and Methods

To address the RQ of this study, a systematic review regarding the PICO components was conducted. The review followed the guidelines set by the PRISMA 2020 Declaration [29]. The steps undertaken to achieve the final result are detailed in Table A1. Prior to that, this procedure was preregistered in the international prospective registers of systematic reviews (PROSPERO) with the identification code CRD42024510781.

### 2.1. Information Sources and Search Strategies

The search began on 30 October 2023, and concluded on 10 February 2024, incorporating records from the final months of 2023. An exhaustive search of English and Spanish scientific literature was carried out. The databases used were Web of Science (WoS), PsycInfo, Scopus, PubMed, PubPsych, CINAHL, ERIC and Dialnet. The search strategies combined different terms depending on the database where they were located. Terms were combined as follows:

- (autis* OR ASD) AND (child* OR student) AND ((canine OR dog) NEAR/2 (therap* OR intervention)) for WoS and PsycInfo;
- (autis* OR ASD) AND (child* OR student) AND (“canine assisted” OR “therapy dog”) for Scopus, PubMed, PubPsych, and CINAHL;
- autism AND dog for ERIC;
- autis* AND perro for Dialnet.

Boolean operators such as AND and OR were integrated, along with proximity operators like NEAR/2. The former was used to retrieve relevant information containing both terms closely related, while maintaining flexibility compared to the restrictive nature of quotation marks. Initially, the most complex search string was applied across all databases. In cases where no results were returned, the search was simplified, either by restricting terms with quotation marks or, for ERIC and Dialnet, by relating terms more broadly. This helped the process to be more sensitive to each database’s browser, what, in turn, was more useful for the consecution of the results.

### 2.2. Eligibility Criteria and Selection Process

To select the most relevant material for this study, the following inclusion and exclusion criteria were applied. The following inclusion criteria were considered (a) all study participants must have been diagnosed with ASD; (b) studies must provide both qualitative information and statistical or quantitative data allowing numerical estimation; (c) studies that are focused on animal-assisted therapy (AAT) and contained a specific section on the effects and results of DAT; (d) participants with commorbid diagnoses, such as ADHD or intellectual disability, as long as they were diagnosed with ASD; (e) Interventions independent of the context or countries where they were conducted. As the exclusion criteria, the following were taken into account (a) studies involving participants older than 18 years old; (b) studies not primarily aimed at evaluating the impact or benefits of DAT; (c) studies lacking empirical evidence; (d) systematic reviews or meta-analyses; (e) doctoral theses; (f) studies analyzing the benefits of living with dogs in a domestic context; (g) documents with no access to their full-texts; (h) documents exclusively addressing the experiences of therapists or family members of individuals with ASD who received DAT.

### 2.3. Data Collection Process and Critical Appraisal

Mendeley was used as the bibliographic reference manager. Additionally, an inter-rater evaluation process was carried out using Covidence, ensuring the adequacy and reliability of the studies for inclusion in the systematic review. Furthermore, the GRADE guidelines were followed to assess the quality of evidence in the selected studies. A protocol to systematically integrate common information from the reviewed articles was implemented. A synthesis of the selected studies was retrieved and presented in a table following the chronological order. The focusing on the following elements: (a) Authors and publication year; (b) Study objective; (c) Participants with ASD: age and gender; (d) Type of study and presence of a control group; (e) Location and timing of the sessions; (f) Instruments used for evaluation and their reliability; and (g) Results obtained.

## 3. Results

### 3.1. Study Selection and Study Characteristics

**Figure 1:**
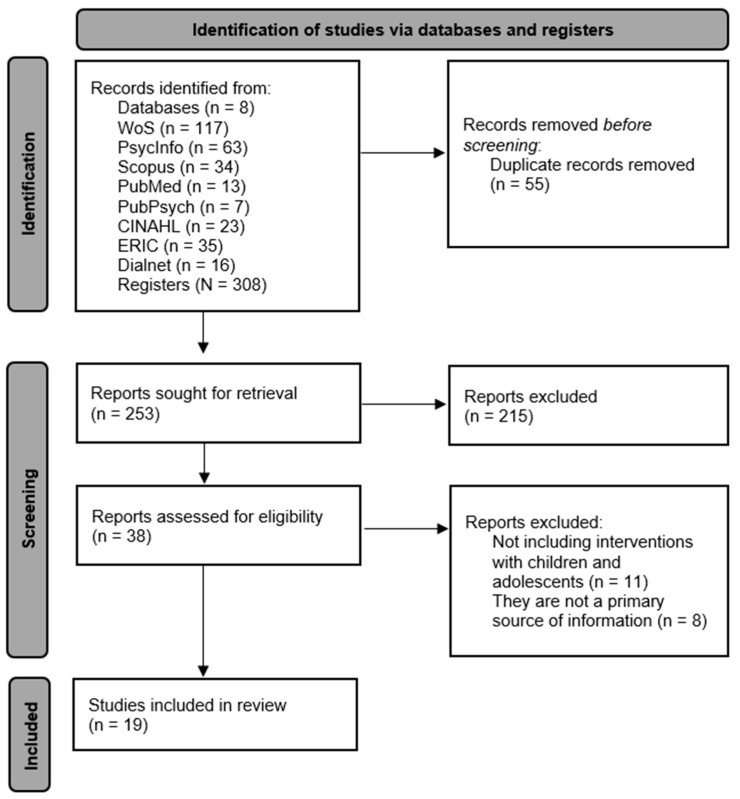
Flowchart regarding PRISMA guidelines.

An initial search yielded 308 results. Regarding the databases in which they were found, 117 appeared in WoS, 63 in PsycInfo, 34 in Scopus, 13 in PubMed, 7 in PubPsych, 23 in CINAHL, 35 in ERIC and 16 in Dialnet. Of them, 55 records appeared in more than one database, so they were eliminated by considering them duplicate. Inclusion and exlusion criteria were applied after reading the abstract. The studies that did not meet the criteria were removed, and during the screening phase, 215 records were eliminated for being irrelevant or not meeting the eligibility criteria. As a result, only 38 documents remained. These were subjected to a more detailed assessment, and ultimately, only 19 studies were deemed suitable for inclusion in the systematic review. The main reasons for discarding documents were, firstly, that they did not involve interventions with children and adolescents. They focused on family or therapist perspectives gathered through questionnaires or interviews instead. Secondly, they were excluded as they were systematic reviews or meta-analyses, which are not considered a primary source of information. The search process and the number of documents identified in each database are presented in Figure 1.

Table 1 outlines the key characteristics of the studies comprising the core of this review (*n* = 19). The majority (95%) are written in English (*n* = 18), and only one in Spanish (Paredes-Ramos et al., 2016). All studies were published within the past 15 years, 63% (*n* = 12) of them released in the last five years.

### 3.2. Synthesis of Results

First, the characteristics of the studies, such as sociodemographic and methodological aspects, will be detailed, followed by an analysis of the results obtained in the interventions.

#### 3.2.1. Sociodemographic Aspects

The interventions were conducted in various geographical regions. Nine studies were carried out in Europe, specifically, one in Germany [48], one in Greece [30], one in the Netherlands [40], one in Poland [42], two in Portugal [38,47], two in Spain [32,37] and one in the United Kingdom [45], accounting for 47% of the sample. In North America, six studies were conducted, one in Canada [34], one in Mexico [44] and four in the United States [36,39,41,43]. In Asia, only two interventions were conducted, one in Hong Kong [46] and one in Israel [33]. In Oceania, two studies were conducted in Australia [31,35].

#### 3.2.2. Sample Size and Participant Characteristics

The 19 studies included a total of 20 interventions, as Dollion et al. [34] carried out two interventions in the same location at different times. The first of them occurred between 2011 and 2014, while the second was conducted in 2020. Regarding sample size, there is considerable variability. The smallest sample consisted of one participant [47], while the largest had 73 participants [33]. In general terms, the sample sizes were quite small, as almost 80% (n = 15) of the studies were conducted with 20 or fewer participants. Only children and young people with ASD meeting the inclusion criteria were considered participants, despite some studies included individuals with other disabilities or ages in their research [38,40,42]. Participant ages, based on the inclusion criteria, ranged from 2 to 14 years old.

One notable aspect is the gender of the participants, with a significant predominance of males over females. Out of 324 participants across all studies, 242 (75%) were boys, 61 (19%) were girls, and 21 (6%) were unspecified. Only Ávila-Álvarez et al. [32] did not report the gender of the participants, although it was noted that the majority were boys.

The selected studies primarily involved students with mild to moderate autism, corresponding to levels 1 and 2 in the DSM-5-TR classification. Only Silva et al. [38] included individuals with more severe autism, while Becker et al. [43] exclusively worked with children diagnosed with high-functioning autism. Some studies provided information on the comorbidities of participants with ASD, such as ADHD [34,37], epilepsy [39], or Tourette syndrome [37], in addition to intellectual disabilities of varying degrees. The participants’ intelligence levels or language acquisition status were not differentiated, as not all studies provided such specific information.

#### 3.2.3. Intervention Settings, Roles of Professionals, and Participants

The interventions were carried out in a variety of settings. Six of them were located in special education schools, five in private foundations, two in public early intervention foundations, two in clinics and hospitals, one in a kindergarten and one at the participants’ homes.

Generally, occupational therapists trained in AAT and special education, the dog’s trainer, and the child or group of children being treated were present. The therapist’s role was to guide and facilitate communication, while the trainer’s role was limited to supervising the dog’s behavior. They intervenied only when necessary for the animal’s well-being or the participant’s safety. In some studies, especially those involving groups, more specialized AAT therapists were present, such as in Tepper et al. [31] and Ben-Itzchak and Zachor [33]. In studies like Ávila-Álvarez et al. [32] and Dollion et al. [34], a family member of the participant could also attend the sessions.

#### 3.2.4. Number and Duration of Sessions

The total number of sessions was unknown in the 32% of the total amount of studies. Specifically, the duration of the intervention was not detailed in the studies by Jorgenson et al. [36], Protopopova et al. [39], and Paredes-Ramos et al. [44]. Additionally, Dollion et al. [34], Germone et al. [41], and Grabowska and Ostrowska [42] did not provide detailed information on intervention duration. Among the 13 studies where the duration was known, it varied widely, ranging from three sessions [38,48] to 32 sessions [33]. Of these, 54% (n = 7) lasted more than 10 sessions, while 46% (n = 6) did not reach this number. Session duration was known in 16 studies (84%), with an average duration of 35 min per session.

Additionally, 78% (n = 15) of the interventions were conducted individually with the participant, while four studies worked in groups [32,33,42,43].

#### 3.2.5. Use of Therapy Dogs

Regarding the therapy dogs, all studies involved animals trained for therapeutic purposes and subjected to veterinary checks. However, Stevenson et al. [45] did not use a dog specifically trained as a therapy dog but worked with the animal and students beforehand to create a safe research environment. Although some studies did not provide this information, the most commonly used breeds were Labrador Retrievers, in seven studies, Golden Retrievers, in two studies, and Border Collies, in two studies, with some studies using mixed-breed dogs.

#### 3.2.6. Evaluation Tools and Data Collection

Regarding the evaluation tools used, a wide variety of instruments to analyze the outcomes of the interventions were employed. Some studies used standardized instruments such as Assessment of Communication and Interaction Skills (ACIS), a scale designed to measure changes in communication and social interaction skills [32,37]; Individual Child Engagement Record-Revised (ICER-R), which assesses the time a child is engaged and interacting with their environment during sessions [32]; and the AAT Flow Sheet, a questionnaire for examining the effects of animal-assisted therapy on social participation [37]. Child Behavior Checklist (CBCL) was also used to analyze participants’ emotional and behavioral issues [40].

Other standardized examples include tools used by Ben-Itzchak and Zachor [33]: Vineland Adaptative Behavior Scales (VABS), for assessing adaptive behavior; Spence Children’s Anxiety Scale (SCAS), for evaluating anxiety; and Social Responsiveness Scale- Second edition (SRS-2), for assessing social behaviors and communication in individuals with ASD, also employed by Becker et al. [43]. These authors also used other standardized measures such as Childhood Autism Rating Scale-Second Edition (CARS-2), for examining autism severity in children; Children’s Depression Inventory 2nd edition (CDI-2), for language development evaluation; Reading the Mind in the Eyes Test (RMET), for assessing emotion and thought interpretation abilities; and Social Language Development Test (SLDT), a measure of social and nonverbal language skills. In addition to the aforementioned standardized tools, other studies opted for non-standardized instruments such as teacher diaries [30], family surveys [42], or teacher questionnaires [45]. Some studies utilized innovative tools like physiological measures to assess salivary cortisol levels [39] or eye-tracking glasses to monitor eye movement and activity in response to stimuli [34].

The interventions were recorded in 73% (*n* = 14) of the studies for later analysis and data collection. Some studies used statistical analysis tools like the Student’s *t*-test to determine significant differences between two variables [32,44]; Cross Recurrence Quantification Analysis (CRQA) to analyze interactions between two elements [40]; and Analysis of Variance (ANOVA) [38,40,48]. Statistical software like SPSS was used in 31% of studies (n = 6). Regarding the reliability of the tools, 21% (n = 4) of studies used Cronbach’s Alpha [33,37,41,43], with internal consistency exceeding 0.7 in all cases. Becker et al. [43] used the Kuder-Richardson 20 (KR20) coefficient to assess the internal consistency of the Social Language Development Test (SLDT) specifically. To evaluate inter-observer agreement and reliability, 31% of studies employed Cohen’s Kappa [30,31,32,40,41,48]. Likewise, interobserver agreement (IOA) was used in two studies [36,39], demonstrating agreement in both, exceeding 90%. Similarly, the studies by Stevenson et al. [45] and Silva et al. [47] use Pearson’s correlation to evaluate agreement between observers. Finally, in two studies, the reliability of the instruments used is not explicitly reported [42,44].

#### 3.2.7. Study Design Types

Regarding the type of studies, there is considerable variety, although in all of them there is intervention involving DAT. Uncontrolled clinical trials are the most used to assess the effectiveness of canine therapies, representing the 47% (*n* = 9) of the 19 studies. They evaluate changes in participants’ behavior after therapies but lack a control group, making it difficult to establish causality [30,31,34,40,42,44,45,48]. Similarly, the study by Silva et al. [38], besides being an uncontrolled clinical trial, conducts its research with a within-subject design, comparing the effects with the same individual receiving the therapy. There are three studies with a quasi-experimental design, one of them with a longitudinal and within-subject design [32], another purely longitudinal [37], and finally, one with a reversal design [36].

The presence of two cross-sectional studies stands out [33,41]. These studies, along with Hill et al. [35], Becker et al. [43], and Fung and Leung [46], are randomized controlled trials, providing high-quality evidence for the research. Finally, there are two pseudo-experimental studies [39], one of which is a single-case design [47].

Only 36% of the studies have a control group (*n* = 7). Two of them feature two distinct groups: an experimental group receiving DAT and a control group receiving conventional therapy [35,43]. In two other studies, a similar situation occurs. There were two distinct groups, one receives DAT before the other, serving as a control group while not receiving the DAT [33,41]. In the study by Fung and Leung [46], an experimental group receives canine therapy, and a control group uses a doll as a substitute for the dog.

Finally, two other studies mention a control group and an experimental group, but the same subjects participate in both. In the case of Protopopova et al. [39], the control group is assigned non-contingent exposure to the dog, while the experimental group receives contingent access to the dog. In contrast, Silva et al. [47] considers that the same participant in their study will serve as a control during sessions involving only the therapist and as experimental when the dog is also included.

#### 3.2.8. Quality of Evidence

The GRADE method was used to assess evidence quality, revealing that 21% of studies were contemplated as high quality [33,41,43,46], 58% were of moderate quality [30,32,34,35,38,40,42,45,47,48] and 21% of low quality [31,36,39,44]. The process for assessing the quality of the evidence and the recommendation for its implementation is presented in Table 2.

#### 3.2.9. Results

Among the 19 studies, 52% (*n* = 10) aimed to study participants’ behavior, social interaction, and communication, and to determine whether or not there was an improvement after DAT [30,31,32,37,41,43,44,45,46,47].

Regarding the results of the studies, the diversity and heterogeneity of the programs applied stand out. However, it is suggested that DAT could have beneficial effects for young people with ASD in 84% (*n* = 16) of the interventions. On the other hand, some studies indicate that the results are inconclusive, as there are significant limitations. For instance, results were not statistically significant enough to affirm that the benefits are due to DAT [31,35] or they were so disparate among participants that no precise conclusion can be reached [36]. Although conclusive results were not obtained, none of them indicate that canine therapy had detrimental effects on participants.

Some of the most common benefits observed after the intervention are related to communication and interaction with the environment. Thus, it is suggested that, after DAT, eye contact is more frequent and lasts longer [30,37,41,44,47], tolerance to physical contact and sensory stimuli increases [30,37,47], and spontaneous imitation and social motivation rise [30,38].

Additionally, there is a notable increase in verbal statements [36,41,46], the initiative to start tasks spontaneously, and greater participation in group activities [30,47]. It is also noteworthy that various studies agree that participants laugh and smile more after interacting with the animal [30,32,44,47]. Some studies indicate an increase in the development of motor skills in young participants. While in Tepper et al. [31], participants showed a greater tendency to remain seated and still in the presence of the dog, the study suggests that the dog may have a calming effect, though this is inconclusive. The research by Grabowska and Ostrowska [42] reinforces this idea, asserting that the animal’s proximity makes children calm and relaxed.

Moreover, studies support the reduction of self-injurious, aggressive, and stereotyped behaviors after therapy assisted with dogs [30,42,47,48]. The study of Ben-Itzchak and Zachor [33], where repetitive and restrictive behaviors increased in intensity, along with the participants’ anxiety, is an exception of that. However, the study itself provides a possible explanation, as this only occurred in the first group receiving canine intervention, coinciding with the start of classes. As the authors explain, students with ASD show high resistance to change, and uncertainty produces significant anxiety for them, which could explain that the increase in these behaviors is not due to canine therapies but to the novelty they face at the beginning of a school year.

Becker et al. [43] argue that incorporating DAT, in addition to bringing improvements in ToM, reduces depressive symptoms in participants, including feelings of isolation and loneliness. In line with this, Paredes-Ramos et al. [44] and Stevenson et al. [45] confirm that after applying the therapies, there was a decrease in children’s isolation and solitary play. Protopopova et al. [39] highlight that the dog serves as a reinforcer for 80% of participants. Dollion et al. [34] describe the dog as a visually attractive companion for children and young people with ASD. Similarly, authors such as Paredes-Ramos et al. [44], Stevenson et al. [45], and Fung and Leung [46] consider the dog to be a communication facilitator, capable of capturing the child’s attention and motivating them to communicate verbally.

## 4. Discussion

The aim of this study was to explore the characteristics of DAT in children and young people with ASD in order to assess the effectiveness and feasibility of its implementation. A total of 308 studies were identified in an initial search, of which 19 met the inclusion criteria and were selected for this work.

The findings of this research indicate DAT, considered a type of complementary therapy, have a positive impact on children and adolescents diagnosed with ASD, particularly in developing communication, social, and interaction skills. Dogs act as reinforcers, stimulating communication and capturing the attention of participants. Additionally, these therapies reduce depressive symptoms, isolation, and stereotyped or restrictive behaviors. This systematic review adds a series of elements enriching the initial general response. The results obtained are consistent with those of other reviews and meta-analyses that have found DAT to be effective in improving various areas of physical and mental health [26,49,50,51,52,53].

Regarding the gender of participants, a marked predominance of male was observed, consistent with the fact that ASD is diagnosed three to four times more frequently in males than females [7]. According to the age of participants, Dollion et al. [34] suggest that it is a critical factor in studying interactions between children with ASD and animals, as older children showed a higher interaction with therapy dogs. Tepper et al. [31] support this notion, indicating that younger children might not benefit as much from animal interactions compared to older children. Conversely, Ben-Itzchak and Zachor [33] argue that younger children tend to show better results in interventions, highlighting the importance of early diagnosis and intervention.

People diagnosed with autism may face significant difficulties in developing social skills and interacting with peers. According to Jackson et al. [54], students with ASD may experience high levels of loneliness, anxiety, and depression, with a high dropout rate before graduation. The observed results indicate emotional benefits in children and adolescents. For instance, in Becker et al. [43], participants experienced increased self-esteem and smiles occurring more frequently. This is particularly relevant because, as Stewart et al. [55] pointed out, individuals with ASD, especially children and adolescents, are more likely to develop depression compared to the general population. Achieving pleasant behaviors that enhance emotional well-being and quality of life might therefore be a primary goal of any therapeutic intervention.

Some studies that did not yield significant results may have been affected by the limited number of sessions. For instance, Griffioen et al. [40] conducted only six sessions. Similarly, Silva et al. [47] noted that a small number of sessions likely hindered the development of an emotional bond with the animal. In Ávila-Álvarez et al. [32], 24 sessions were conducted, with improvements observed after the twelfth session. Consistent with this, Flujas-Contreras et al. [56] found that interventions exceeding 12 sessions for individuals with ASD demonstrated a larger effect size. Nevertheless, 32% of the studies lacked detailed information about the total duration of the intervention, introducing a bias that restricts the principle of scientific replicability.

On the other hand, it is noteworthy that during the search process, a systematic review was identified that appears to study the same field as the present review [28]. After analyzing this work, the authors decided to continue with the search because, despite having a very similar title, there are other differential aspects that will be discussed in detail in Table A2. Broadly speaking, after evaluating the contribution of Poveda Gómez et al. [28], it is worth mentioning that their review covers up to April 2021, using the following databases: WoS, PubMed, Scopus, and CINAHL. They selected 19 articles and their synthesis examines the objectives, participant samples, the intervention performed, and the results found. In this way, studies are classified into two types: those exploring the benefits of assistance dogs at home and those of DAT interventions for children with ASD. Also, studies evaluating family perspectives to understand their stress levels, the views of the care therapist, or attitudes towards therapy dogs, among others, were included. These contributions are very interesting for the field of study but not considered in the current study.

### 4.1. Limitations

The success of interventions may have been influenced by limiting factors. For instance, many studies selected participants who already displayed an interest in or attraction to dogs, excluding children with allergies, fear, or aggressive behaviors toward animals, as seen in Griffioen et al. [40]. Additionally, some participants already lived with dogs as pets at home, resulting in heterogeneous starting points. However, Protopopova et al. [39] found that even children without an initial preference for dogs benefitted from the animal’s role as a reinforcer in educational behaviors and school tasks. Professionals should consider the personal characteristics of participants before implementing interventions.

Another limitation is the need for replication of studies with larger participant samples to ensure the generalization of results [34,35,40,43,45,46]. Jorgenson et al. [36] highlighted the absence of standardized treatments and replicable results in the literature. Additionally, some studies lacked a control group, such as Ávila-Álvarez et al. [32], Ávila-Álvarez et al. [37], and Grabowska & Ostrowska [42], making it difficult to attribute changes solely to DAT. Finally, studies like Ávila-Álvarez et al. [32] and Silva et al. [38] emphasized the need to verify whether observed results persisted over time after the intervention.

Using the GRADE methodology, two inconclusive studies were classified as low-quality. These studies indicate that the obtained results were not significant, preventing definitive conclusions. Given the low quality of these studies, the true effect of the research could differ substantially from the estimated effect. As mentioned earlier, only four studies in the systematic review showed a high quality. More resources should be allocated to improve the quality of such research, as higher methodological quality leads to more robust and evidence-based results. Following GRADE guidelines, a conditional recommendation is made for implementing DAT in children and adolescents. While evidence supports their efficacy, factors such as participant preferences, age, and intervention duration should be evaluated due to limitations and biases that may affect the validity, reliability, and generalizability of the results.

### 4.2. Practical Implications

Regarding practical implications, a DAT program could be developed in specific units within mainstream schools focusing on Communication and Language, targeting preschool and primary students with ASD. In their review, Poveda Gómez et al. [28] noted a lack of dog-assisted therapy interventions in educational settings. However, this review identifies six studies conducted in special education schools [30,33,40,45,46], one in a preschool [42], and another mentioning a school setting [43].

Intervention programs may include a minimum of 12 sessions, as evidence suggests that effectiveness increases from this threshold. Implementation would require trained professionals and the supervision of canine behavior specialists to ensure the safety and well-being of both participants and animals.

### 4.3. Future Research

Future research should focus on analyzing the influence of participant age on interactions with therapy dogs to determine the ages at which interventions yield the best results. Long-term benefits should also be evaluated. Furthermore, studies comparing individual versus group therapy modalities would be insightful.

Conducting interventions with larger samples and including both control and experimental groups would strengthen the evidence base. Additionally, a systematic review in the coming years would update the trends observed in this field, given the notable increase in related studies over the past five years.

## 5. Conclusions

The evidence presented in this review strongly supports the effectiveness of DAT as a promising approach to enhancing the well-being and development of children and adolescents with ASD. The positive impact of DAT on social, emotional, and behavioral outcomes highlights its potential as a valuable therapeutic tool within educational settings. Despite certain limitations, such as variability in study designs and number of sessions, the overall body of evidence provides a robust foundation for recommending DAT as a complementary intervention in promoting the holistic development of children and adolescents with ASD. However, it is crucial to acknowledge that a careful, individualized evaluation of each participant’s unique characteristics and needs is essential to maximize the potential benefits of AAT. Personalized approaches like this will ensure that the therapy is tailored to the specific challenges and strengths of each child or adolescent, which is key to its success. Given the growing interest in it, this therapeutic approach has the potential to become a common practice, making a significant contribution to the development of children and adolescents with ASD and other developmental disorders.

## Figures and Tables

**Table 1:** Synthesis of the selected studies.

Authors and Publication Year	Objective	Sample N Age (Min.–Max.) Gender (Women/Men)	Study Design CG	Location Session’s Timing	Instruments Reliability	Results
Karpoutzaki et al. (2023) [30]	To investigate the effectiveness of a therapy dog’s presence on the social behavior and communication of students with ASD	3 10–12 1/2	Non-controlled clinical trial. CG = No	Special education school (Greece). 6 individual sessions, 30 min each	Questionnaire for family members and teacher’s diary; microanalysis of video recordings. RCI (*z* = ±1.96)	Higher tolerance to sensory stimuli and physical contact. Higher eye contact and social responsiveness. Lower self-injurious, aggressive, and stereotypical behaviors
Tepper et al. (2022) [31]	To assess the impact of DAT on social communication skills, executive functions, and motor skills in children with ASD	16 2–4 7/9	Non-controlled clinical trial. CG = No	Early intervention service (Australia). 18 group sessions, 1 h 30 min each	Interval recording and SPSS 23.0 analysis. (*k* = 0.8; *p* < 0.001).	Higher tendency to remain calm and seated during active dog participation. Inconclusive overall results
Ávila-Álvarez et al. (2022) [32]	To assess the feasibility of early implementation of DAT for children with neurodevelopmental disorders and analyze social functioning changes	21 ** 2–6 NR	Quasi-experimental, longitudinal within-subject design. CG = No	Public rehabilitation and early care unit (Spain). 24 individual sessions, 45 min each	ACIS, ICER-R, SPSS 27. *k* (*p* < 0.05)	Higher communication and interaction. Higher engagement during sessions, with socially appropriate behaviors. Higher frequency of interaction with adults
Ben-Itzchak & Zachor (2021) [33]	To assess the effectiveness of a DAT program on adaptive skills, autism severity, and anxiety in children with ASD	73 2–7 12/61	Cross-sectional, randomized controlled trial. CG = Yes	Special education school (Israel). 32 group sessions, 20–45 min each	SRS-2, VABS, SCAS, DTI. (SCASS’ *α*: 0.86–0.94)	An increase in their social, communication and motor skills. An increase in their repetitive and restricted behavior (which decreased after the second semester)
Dollion et al. (2021) [34]	Two studies exploring the visual attention of children with ASD and their behavior during the first interaction with a service dog	Study 1: 16 4–13 2/14 Study 2: 6 6–14 3/3	Non-controlled clinical trial. CG = No	Mira Foundation (Canada). Individual sessions	Video analysis, eye-tracking glasses, semi-structured interviews with families and post-intervention. (*k* = 0.81)	Higher visual attention to the dog, especially its head. Increased child-initiated free interaction with petting. Closer interaction with the animal as age increases, with more attention given to the dog and family
Hill et al. (2020) [35]	To assess the impact of occupational therapy assisted with dogs on concentration behaviors and goal achievement in autistic children	22 4–6 6/16	Randomized, controlled pilot clinical trial CG = Yes	Animal-assisted psychology clinic (Australia). 9 individual sessions, 1 h each	Video coding, SPSS 25. Fidelity checklist for intervention (97.93%)	Positive trend in concentration and goal achievement behavior (not statistically significant). Higher performance and perceived satisfaction when parents participated in therapy
Jorgenson et al. (2020) [36]	To evaluate the preference and effectiveness of using a therapy dog as reinforcement to increase verbal statements	5 3–8 1/4	Quasi-experimental reversal design. CG = No	Outpatient clinic (the USA). Individual sessions, twice per week	Video recording, MSWO. IOA (91–99%, per participant)	Higher verbal statements in two children with ASD and higher social interaction in one participant. Results varied between subjects
Ávila- Álvarez et al. (2020) [37]	To explore the feasibility of early intervention using therapy dogs and examine their impact on communicative and social interaction skills	19 2–6 6/13	Quasi-experimental, longitudinal. CG = No	Public rehabilitation and early care unit (Spain). 9 individual sessions, 20 min each	ACIS, AAT Flow Sheet, SPSS 22.0. Inter-rater agreement (0.98), (*α* = 0.78), *k* (*p* < 0.05)	Increased use of body in their social interactions (e.g., head directioning), higher social relationship skills (e.g., maintaining attention) and higher frequency of visual, verbal and physical contact with the animal
Silva et al. (2020) [38]	To examine the feasibility of using DAT as an effective method to stimulate spontaneous imitation in children and adults with severe ASD	10 ** 5–8 0/10	Non-controlled within-subject clinical trial. CG = No	Participants’ homes (Portugal). 3 individual sessions	Coding, pre- and post-tests with data analysis. ANOVA, SPSS 24. (*k* = 0.92)	Spontaneous imitation and social motivation after free interaction with the dog
Protopopova et al. (2019) [39]	To evaluate how the presence of a therapy dog affects children with ASD through contingent and non-contingent access during educational tasks	5 7–11 1/4	Pseudo-experimental design. CG = Yes	Classroom (the USA). Individual sessions, 30 min each	Video recording, behavioral and physiological measures (cortisol levels). IOA (96.8%)	Better behavior during academic tasks when the dog was presented contingently. Higher social interaction and bonding when the dog was presented non-contingently
Griffioen et al. (2019) [40]	To analyze behavioral synchrony during DAT for children with ASD and Down syndrome	5 ** 11–13 1/4	Uncontrolled clinical trial. CG = No	Special education school (the Netherlands). 6 individual sessions, 30 min each	Video recording, CBCL (families), CRQA, ANOVA. Inter-rater agreement (>80%)	Higher behavioral synchrony between child and dog. Lower behavioral problems (although not statistically significant)
Germone et al. (2019) [41]	To investigate the benefits of dog-assisted activities on social behaviors of psychiatrically hospitalized youth with ASD	47 6–8 8/39	Randomized, cross-sectional pilot clinical trial. CG = Yes	Pediatric hospital in Colorado (the USA). Individual sessions, 20 min each	Video recording, coders, OHAIRE-3. (*α* = 0.77), (*k* = 0.87)	Increased social communication behaviors and increased positive facial expressions, greater speech, use of gestures, and gaze toward adults and peers
Grabowska & Ostrowska (2018) [42]	To evaluate the effectiveness of DAT as a complementary rehabilitation method for children with developmental disabilities	12 ** 4–9 5/7	Non-controlled clinical trial. GC = No	School (Poland). One weekly group session, 30 min each	Non-standardized family survey, statistical software. NR	Children’s social and emotional domains improved. Icreased emotional regulation. Dogs showed a calming influence, and reduced frequency of aggressive behaviors
Becker et al. (2017) [43]	To evaluate the effectiveness of DAT on social skills in a young group with ASD	31 8–14 3/28	Randomized, controlled clinical trial. CG =Yes	School (the USA). 12 group sessions, 1 h each	CARS-2 (internal reliability = 0.93), CDI-2 (*α* = 0.67–0.91), RMET, SLDT (KR20 coefficient = 0.93), SRS-2 (*α* = 0.85)	Improvement in ToM, decreased feelings of isolation and depressive symptoms and maintenance of social skills.
Paredes-Ramos et al. (2016) [44]	To evaluate the impact that a therapy dog can have on the behavior of children with ASD and in the interaction with the therapist	5 6–8 0/5	Non-controlled clinical trial. CG = No	CEEDA therapy center (Mexico). Individual sessions, 20 min each	Visual preference test. Behavioral and statistical analysis: t Student. Video recording. NR	Increase in laughter and proportion of successful interactions with the therapist. Higher visual contact and requests to play with the dog compared to the therapist. Decrease in the duration of solitary playing
Stevenson et al. (2015) [45]	To determine if DAT can motivate students with ASD to increase social interaction and engagement with their teacher	3 7–13 0/3	Non-controlled clinical trial. CG = No	Special education school (the UK). 5 individual sessions, 20 min each	Semi-standardized approach, qualitative observations and teacher questionnaires. Inter-rater reliability varied between 45% and 93% across categories (Pearson’s correlation).	Increase in significant social interactions with the dog and teacher. Decrease in solitary or repetitive behaviors
Fung & Leung (2014) [46]	To provide evidence of the effectiveness of incorporating therapy dogs to facilitate social interaction in children with ASD in a structured play context	10 7–10 2/8	Randomized, controlled clinical trial. CG = Yes	Special education school (Hong Kong). 14 individual sessions, 20 min each	Quantitative social behavior observation, coding system, Mann-Whitney U test. IOA (97.9–99.3%)	Statistically significant increase in speech in the experimental group. Therapy dog positively impacted on language production
Silva et al. (2011) [47]	To provide quantitative evidence on the potential of dogs to positively modulate the behavior of children with ASD	1 12 0/1	Pseudo-experimental single-case study. GC = Yes	At their current treatment settings (Portugal). 12 individual sessions, 45 min each	Video recording and software coding. Pearson correlation (r = 0.9)	More frequent and longer-lasting positive behaviors (e.g., smiling, positive physical contact). Less frequent and shorter-lasting negative behaviors (e.g., aggressive manifestations)
Prothmann et al. (2009) [48]	To provide different types of complex social stimuli to children with ASD in order to observe their preferences and responsiveness in a real-life situation	14 6–14 3/11	Non-controlled clinical trial. CG = No	A clinic (Germany). 3 individual sessions, 20 min	Video recording. Coding software to analyze behavior. ANOVA, SPSS 12.0. (*k* = 0.83)	More frequent and longer-lasting interaction with the dog. Higher interest in social relationships. Lower self-stimulatory behavior, such as stereotypies, during sessions with the dog

Note. CG: control group; ASD: Autism Spectrum Disorder; RCI: Reliable Change Index; *z*: Z value; DAT: Dog-assisted therapy; SPSS: Statical Package for the Social Sciences; *k*: Cohen’s Kappa; NR: Not reported; ACIS: Assessment of Communication and Interaction Skills; ICER-R: Individual Child Engagement Record-Revised; SRS-2: Social Responsiveness Scale-second edition; VABS: Vineland Adaptative Behavior Scales; SCAS: Spence Children’s Anxiety Scale; DTI: “Dog Time”; Chronbach’s alpha: *α*; MSWO: Multiple Stimulus Without Replacement; IOA: interobserver agreement; AAT: Animal-assisted therapy; ANOVA: Análisis of variance; CBCL: Child Behavior Checklist; CRQA: Cross-Recurrence Quantification Analysis; OHAIRE-3: Observation of Human-Animal Interaction for Research 3rd edition; CARS-2: Childhood Autism Rating Scale-Second Edition; CDI-2: Children’s Depression Inventory 2nd Edition; RMET: Reading the Mind in the Eyes Test y SLDT: Social Language Development Test; KR20: Kuder-Richardson 20 coefficient. ** Only participants with ASD aged between 0–18 years are accounted in this table. Individuals with other disabilities or age ranges can be part of the sample of the study, although not included in the table.

**Table 2:** Results of GRADE method implementation among the 19 studies included in the review.

Authors and Publication Year	Study Design	Study Limitations (Risk of Bias)	Inconsistency	Indirect Evidence	Imprecision	Other Considerations	Quality
Karpoutzaki et al. (2023) [30]	Non-controlled clinical trial	Serious limitations ^1^	Not serious	Not serious	Serious ^2^	Not detected	⨁⨁⨁◯ Moderate
Tepper et al. (2022) [31]	Non-controlled clinical trial	Serious limitations ^1^	Serious ^3^	Serious ^4^	Not serious	Not detected	⨁⨁◯◯ Low
Ávila-Álvarez et al. (2022) [32]	Quasi-experimental, longitudinal within-subject design	Serious limitations ^1,5^	Not serious	Not serious	Not serious	Not detected	⨁⨁⨁◯ Moderate
Ben-Itzchak & Zachor (2021) [33]	Cross-sectional, randomized controlled trial	No serious limitations	Not serious	Not serious	Not serious	Not detected	⨁⨁⨁⨁ High
Dollion et al. (2021) [34]	Non-controlled clinical trial	Serious limitations ^1^	Serious ^6^	Not serious	Not serious	Not detected	⨁⨁⨁◯ Moderate
Hill et al. (2020) [35]	Randomized, controlled pilot clinical trial	Serious limitations ^7,8^	Serious ^3^	Not serious	Not serious	Not detected	⨁⨁⨁◯ Moderate
Jorgenson et al. (2020) [36]	Quasi-experimental reversal design	Serious limitations ^1^	Serious ^3,6^	Not serious	Serious ^2^	Not detected	⨁⨁◯◯ Low
Ávila- Álvarez et al. (2020) [37]	Quasi-experimental, longitudinal	Serious limitations ^1,8^	Not serious	Not serious	Not serious	Not detected	⨁⨁⨁◯ Moderate
Silva et al. (2020) [38]	Non-controlled within-subject clinical trial	Serious limitations ^1,8^	Not serious	Not serious	Not serious	Not detected	⨁⨁⨁◯ Moderate
Protopopova et al. (2019) [39]	Pseudo-experimental design	Serious limitations ^9^	Serious ^6^	Not serious	Serious ^2^	Not detected	⨁⨁◯◯ Low
Griffioen et al. (2019) [40]	Uncontrolled clinical trial	Serious limitations ^1,8^	Not serious	Not serious	Serious ^2^	Not detected	⨁⨁⨁◯ Moderate
Germone et al. (2019) [41]	Randomized, cross-sectional pilot clinical trial	No serious limitations	Serious ^6^	Not serious	Not serious	Not detected	⨁⨁⨁⨁ High
Grabowska & Ostrowska (2018) [42]	Non-controlled clinical trial	Serious limitations ^1,8,10^	Not serious	Not serious	Not serious	Not detected	⨁⨁⨁◯ Moderate
Becker et al. (2017) [43]	Randomized, controlled clinical trial	No serious limitations	Not serious	Not serious	Not serious	Not detected	⨁⨁⨁⨁ High
Paredes-Ramos et al. (2016) [44]	Non-controlled clinical trial	Serious limitations ^1,8,10^	Serious ^6^	Not serious	Serious ^2^	Not detected	⨁⨁◯◯ Low
Stevenson et al. (2015) [45]	Non-controlled clinical trial	Serious limitations ^1,8^	Not serious	Not serious	Serious ^2^	Not detected	⨁⨁⨁◯ Moderate
Fung & Leung (2014) [46]	Randomized, controlled clinical trial	No serious limitations	Not serious	Not serious	Not serious	Not detected	⨁⨁⨁⨁ High
Silva et al. (2011) [47]	Pseudo-experimental single-case study	Serious limitations ^1^	Not serious	Not serious	Serious ^2^	Not detected	⨁⨁⨁◯ Moderate
Prothmann et al. (2009) [48]	Non-controlled clinical trial	Serious limitations ^1,8^	Not serious	Not serious	Not serious	Not detected	⨁⨁⨁◯ Moderate
Recommendation							
Source of recommendation against the intervention	Conditional recommendation against the intervention	Conditional recommendation for the intervention or the comparison		Conditional recommendation for the intervention		Strong recommendation for the intervention	
				X			

^1^ Absence of a control group. ^2^ Limited sample size (<10 participants). ^3^ Non-significant results. ^4^ Information inferred that does not derive directly from the research question. ^5^ The study was interrupted by COVID-19. ^6^ Duration of intervention not specified. ^7^ “Ceiling effect”: very good results reported in both groups, no significant differences. ^8^ Intervention duration (<12 sessions). ^9^ Incomplete follow-up (dropouts). ^10^ Reliability of the instruments used is unknown.

## Abbreviations

DAT	Dog-assisted therapy
ASD	Autism Spectrum Disorder
WoS	Web of Science
DSM	Diagnostic and Statistical Manual of Mental Disorders
APA	American Psychiatric Association
WHO	World Health Organization
ToM	Theory of Mind
IAHAIO	International Association of Human-Animal Interaction Organizations
AAI	Animal-assisted interventions
RQ	Research Question
PICO	Patients/Problem, Intervention, Comparison, Outcomes
PRISMA	Preferred Reporting Items for Systematic reviews and Meta-Analyses
PROSPERO	International prospective registers of systematic reviews
AAT	Animal-assisted therapy
GRADE	Grading of Recommendations Assessment, Development and Evaluation
ACIS	Assessment of Communication and Interaction Skills
ICER-R	Individual Child Engagement Record-Revised
CBCL	Child Behavior Checklist
VABS	Vineland Adaptative Behavior Scales
SCAS	Spence Children’s Anxiety Scale
SRS-2	Social Responsiveness Scale-second edition
CARS-2	Childhood Autism Rating Scale-Second Edition
CDI-2	Children’s Depression Inventory
RMET	Reading the Mind in the Eyes Test
SLDT	Social Language Development Test
CRQA	Cross-Recurrence Quantification Analysis
ANOVA	Analysis of variance
KR20	Kuder-Richardson 20 coefficient
IOA	Interobserver agreement

## Appendix A

**Table A1:** PRISMA checklist and the page in which each item appears in the text.

Items	No	Yes	Page	NA
TITLE				
1. Title		X	1	
ABSTRACT				
2. Structured abstract		X	1	
INTRODUCTION				
3. Rationale		X	1	
4. Objectives		X	3	
METHODS				
5. Eligibility criteria		X	4	
6. Information sources		X	4	
7. Search strategy		X	4	
8. Study selection process		X	4	
9. Data extraction process		X	4	
10. List of data		X	4	
11. Risk of bias		X	4	
12. Effect measures				X
13. Synthesis methods				X
14. Assessment of publication bias				X
15. Certainty assessment				X
RESULTS				
16. Study selection		X	5	
17. Study characteristics		X	5	
18. Risk of bias		X	12	
19. Results of individual studies		X	6	
20. Results of synthesis		X	10	
21. Publication bias				X
22. Certainty of evidence		X		
DISCUSSION				
23. Discussion		X	15	
OTHER INFORMATION				
24. Registration and protocol		X	3	
25. Funding		X	17	
26. Conflict of interest		X	17	
27. Availability of data				X

Note. NA: Not applicable.

## Appendix B

**Table A2:** Comparative summary between the work of by Poveda-Gómez et al. (2021) and the current study.

Items	Poveda-Gómez et al. (2021)	The Current Study
Temporality	From 2000 to April 2021	No lower temporality limit. Upper temporality limit is 2023
Databases	WoS, PubMed, Scopus, and CINAHL	WoS, PubMed, Scopus, CINAHL, PubPsych, ERIC, PsycInfo and Dialnet
Selected studies	19. Studies of DAT for children and youth with ASD (n = 11) and studies investigating the benefits of assistance dogs in the home (n = 8)	19. Studies of DAT for children and adolescents with ASD
Eligibility criteria differences	Included the articles where the study population consisted of school-age children with ASD or those responsible for the intervention	Excluded documents exclusively addressing the experience of therapists or family members of people with ASD who have received DAT and studies analyzing the benefits of living with dogs in a family context, focusing on canine therapies rather than the presence of dogs as pets or assistance dogs
Information provided in their results	Authors, objectives, sample, intervention and results	Authors and year, sample (age and gender), study type and control group presence, location and timing, instruments and reliability and results
Location of interventions	No interventions using therapy dogs in educational settings were found	Six studies conducted in special education schools (Ben-Itzchak & Zachor, 2021; Fung & Leung, 2014; Griffioen et al., 2019; Karpoutzaki et al., 2023; Stevenson et al., 2015), one intervention conducted in a preschool (Grabowska & Ostrowska, 2018) and another study mentioning school as the location (Becker et al., 2017)
Structure of the analysed aspects in the studies	Sociodemographic aspects: Gender of participants. Participants’ ages. Diagnostic comorbidities.	Sociodemographic aspects: Geographic location. Sample size and participants’ ages. Gender of participants. Gradation of ASD. Diagnostic comorbidities.
	Methodological aspects: Location of intervention. Duration and number of sessions (not specified). Dog breeds. Study objectives.	Methodological aspects: Location of intervention. Agents present during intervention. Duration and number of sessions. Group and individual interventions. Dog breeds. Instruments used and their reliability. Study design. Control group.
	Results: DAT benefits. Consideration of AAT.	Results: Study objectives. Benefits and results of DAT.
	Discussion: Future research lines. Limitations. Practical implications.	Discussion: Influencing factors (e.g., canine attraction, age, number of sessions, etc.). Limitations. Practical implications. Future research lines. Conclusion.

## References

[1] American Psychiatric Association (2023). Manual Diagnóstico y Estadístico de los Trastornos Mentales.

[2] Castillo Bautista J.C., Suricalday A.S. (2023). Intervenciones eficaces para la mejora de las habilidades sociales en personas con trastorno del espectro autista de alto funcionamiento: Una revisión sistemática. Bordón Rev. Pedagog..

[3] World Health Organization (2022). Autism. https://www.who.int/news-room/fact-sheets/detail/autism-spectrum-disorders.

[4] Portilla C. (2023). Trastorno del espectro autista. Rev. Psicol..

[5] Salari N., Rasoulpoor S., Rasoulpoor S., Shohaimi S., Jafarpour S., Abdoli N., Khaledi-Paveh B., Mohammadi M. (2022). The global prevalence of autism spectrum disorder: A comprehensive systematic review and meta-analysis. Ital. J. Pediatr..

[6] Talantseva O.I., Romanova R.S., Shurdova E.M., Dolgorukova T.A., Sologub P.S., Titova O.S., Kleeva D.F., Grigorenko E.L. (2023). The global prevalence of autism spectrum disorder: A three-level meta-analysis. Front. Psychiatry.

[7] Montagut Asunción M., Más Romero R.M., Fernández Andrés M.I., Pastor Cerezuela G. (2018). Influencia del sesgo de género en el diagnóstico de trastorno de espectro autista: Una revisión. Escr. Psicol..

[8] Navarro-Pardo E., López-Ramón M.F., Alonso-Esteban Y., Alcantud-Marín F. (2021). Diagnostic tools for autism spectrum disorders by gender: Analysis of current status and future lines. Children.

[9] Fernández López A. (2019). Terapia asistida con animales en pacientes con trastorno del espectro autista: Cuidados de enfermería. Conoc. Enferm..

[10] Hervás Zúñiga A., Balmaña N., Salgado M. (2017). Los trastornos del espectro autista (TEA). Pediatría Integral.

[11] Begeer S., Malle B.F., Nieuwland M.S., Keysar B. (2010). Using theory of mind to represent and take part in social interactions: Comparing individuals with high-functioning autism and typically developing controls. Eur. J. Dev. Psychol..

[12] Paula-Pérez I., Artigas-Pallarés J. (2020). La intolerancia a la incertidumbre en el autismo. Medicina.

[13] Calderón R. (2019). La intervención en el trastorno del espectro autista en las alteraciones en intersubjetividad y la teoría de la mente. Educación.

[14] Antshel K.M., Russo N. (2019). Autism spectrum disorders and ADHD: Overlapping phenomenology, diagnostic issues, and treatment considerations. Curr. Psychiatry Rep..

[15] Genovese A., Butler M.G. (2020). Clinical assessment, genetics, and treatment approaches in autism spectrum disorder (ASD). Int. J. Mol. Sci..

[16] Ona H.N., Larsen K., Nordheim L.V., Brurberg K.G. (2020). Effects of pivotal response treatment (PRT) for children with autism spectrum disorders (ASD): A systematic review. Rev. J. Autism Dev. Disord..

[17] Urinovsky M.G., Cafiero P.J. (2022). Tratamientos alternativos y/o complementarios en pacientes con trastorno del espectro autista. Med. Infant..

[18] International Association of Human-Animal Interaction Organizations (IAHAIO) (2018). The IAHAIO White Paper 2014, Updated for 2018: Definitions for Animal Assisted Intervention and Guidelines for Wellness of Animals Involved in AAI. https://iahaio.org/best-practice/white-paper-on-animal-assisted-interventions/.

[19] Dalton K.R., Waite K.B., Ruble K., Carroll K.C., DeLone A., Frankenfield P., Serpell J.A., Thorpe R.J., Morris D.O., Agnew J. (2020). Risks associated with animal-assisted intervention programs: A literature review. Complement. Ther. Clin. Pract..

[20] Bert F., Gualano M.R., Camussi E., Pieve G., Voglino G., Siliquini R. (2016). Animal assisted intervention: A systematic review of benefits and risks. Eur. J. Integr. Med..

[21] Jalongo M.R., Guth L.J. (2023). Animal-assisted counseling for young children: Evidence base, best practices, and future prospects. Early Child. Educ. J..

[22] Kamioka H., Okada S., Tsutani K., Park H., Okuizumi H., Handa S., Oshio T., Park S.-J., Kitayuguchi J., Abe T. (2014). Effectiveness of animal-assisted therapy: A systematic review of randomized controlled trials. Complement. Ther. Med..

[23] Lindström Nilsson M., Funkquist E.L., Edner A., Engvall G. (2020). Children report positive experiences of animal-assisted therapy in paediatric hospital care. Acta Paediatr..

[24] Vegue Parra E., Hernández Garre J.M., Echevarría Pérez P. (2021). Benefits of dog-assisted therapy in patients with dementia residing in aged care centers in Spain. Int. J. Environ. Res. Public Health.

[25] Paredes-Ramos P., Pérez-Pouchoulén M., García-Bañuelos P., Martínez-Conde R., Rioux M., Manzo J., Coria-Avila G. (2012). El uso del perro en el tratamiento del trastorno espectro autista. Rev. Electrónica Eneurobiología.

[26] Rodríguez-Martínez M.D.C., De la Plana Maestre A., Armenta-Peinado J.A., Barbancho M.Á., García-Casares N. (2021). Evidence of animal-assisted therapy in neurological diseases in adults: A systematic review. Int. J. Environ. Res. Public Health.

[27] Hüsgen C.J., Peters-Scheffer N.C., Didden R. (2022). A Systematic Review of Dog-Assisted Therapy in Children with Behavioural and Developmental Disorders. Adv. Neurodev. Disord..

[28] Poveda Gómez M., Marín Suelves D., Navarro Sánchez S. (2021). Intervenciones asistidas con perros en alumnado con Trastorno del Espectro Autista. Una revisión de la literatura. REIDOCREA.

[29] Page M.J., McKenzie J.E., Bossuyt P.M., Boutron I., Hoffmann T.C., Mulrow C.D., Shamseer L., Tetzlaff J.M., Akl E.A., Brennan S.E. (2021). Declaración PRISMA 2020: Una guía actualizada para la publicación de revisiones sistemáticas. Rev. Esp. Cardiol..

[30] Karpoutzaki H., Markodimitraki M., Kypriotaki M., Charitaki G. (2023). El impacto de un programa de perros de terapia en las habilidades sociales, la comunicación y las dificultades de comportamiento de los niños con TEA. Educ. Preesc. Primaria.

[31] Tepper D.L., Landry O., Howell T.J., Stephens D., Molina J., Bennett P.C. (2022). Therapy dogs for children with autism spectrum disorder: Impacts of active versus passive dog engagement. Hum. Anim. Interact. Bull..

[32] Ávila-Álvarez A., Alonso-Bidegain M., Ramos-Veiguela D., Iglesias-Jove E., De-Rosende-Celeiro I. (2022). Changes in social functioning and engagement during canine-assisted intervention for children with neurodevelopmental disorders in the context of an early intervention service. Res. Dev. Disabil..

[33] Ben-Itzchak E., Zachor D.A. (2021). Dog training intervention improves adaptive social communication skills in young children with autism spectrum disorder: A controlled crossover study. Autism.

[34] Dollion N., Toutain M., François N., Champagne N., Plusquellec P. (2021). Visual Exploration and Observation of Real-Life Interactions Between Children with ASD and Service Dogs. J. Autism Dev. Disord..

[35] Hill J., Ziviani J., Driscoll C., Teoh A.L., Chua J.M., Cawdell-Smith J. (2020). Canine Assisted Occupational Therapy for Children on the Autism Spectrum: A Pilot Randomised Control Trial. J. Autism Dev. Disord..

[36] Jorgenson C.D., Clay C.J., Kahng S. (2020). Evaluating preference for and reinforcing efficacy of a therapy dog to increase verbal statements. J. Appl. Behav. Anal..

[37] Ávila-Álvarez A., Alonso-Bidegain M., De-Rosende-Celeiro I., Vizcaíno-Cela M., Larrañeta-Alcalde L., Torres-Tobío G. (2020). Improving social participation of children with autism spectrum disorder: Pilot testing of an early animal- assisted intervention in Spain. Health Soc. Care Community.

[38] Silva K., Lima M., Fafiães C., Sinval J., de Sousa L. (2020). Preliminary test of the potential of contact with dogs to elicit spontaneous imitation in children and adults with severe autism spectrum disorder. Am. J. Occup. Ther..

[39] Protopopova A., Matter A.L., Harris B.N., Wiskow K.M., Donaldson J.M. (2020). Comparison of contingent and noncontingent access to therapy dogs during academic tasks in children with autism spectrum disorder. J. Appl. Behav. Anal..

[40] Griffioen R.E., van der Steen S., Verheggen T., Enders-Slegers M.J., Cox R. (2020). Changes in behavioural synchrony during dog-assisted therapy for children with autism spectrum disorder and children with Down syndrome. J. Appl. Res. Intellect. Disabil..

[41] Germone M.M., Gabriels R.L., Guérin N.A., Pan Z., Banks T., O’Haire M.E. (2019). Animal-assisted activity improves social behaviors in psychiatrically hospitalized youth with autism. Autism.

[42] Grabowska I., Ostrowska B. (2018). Evaluation of the effectiveness of canine assisted therapy as a complementary method of rehabilitationin disabled children. Physiother. Q..

[43] Becker J.L., Rogers E.C., Burrows B. (2017). Animal-assisted Social Skills Training for Children with Autism Spectrum Disorders. Anthrozoös.

[44] Paredes-Ramos P., Gutiérrez-Delfín A., Ortiz-Jiménez X., Carrasco-García A., Perez-Pouchoulén M., Coria-Ávila G.A. (2016). La presencia de un perro mejora la interacción de niños con trastorno del espectro autista y su terapeuta. Rev. Electrónica Eneurobiología.

[45] Stevenson K., Jarred S., Hinchcliffe V., Roberts K. (2015). Can a dog be used as a motivator to develop social interaction and engagement with teachers for students with autism?. Support Learn..

[46] Fung S., Leung A.S. (2014). Pilot Study Investigating the Role of Therapy Dogs in Facilitating Social Interaction among Children with Autism. J. Contemp. Psychother..

[47] Silva K., Correia R., Lima M., Magalhães A., de Sousa L. (2011). Can dogs prime autistic children for therapy? Evidence from a single case study. J. Altern. Complement. Med..

[48] Prothmann A., Ettrich C., Prothmann S. (2009). Preference for, and Responsiveness to, People, Dogs and Objects in Children with Autism. Anthrozoös.

[49] Feng Y., Lin Y., Zhang N., Jiang X., Zhang L. (2021). Effects of animal-assisted therapy on hospitalized children and teenagers: A systematic review and meta-analysis. J. Pediatr. Nurs..

[50] Jain B., Syed S., Hafford-Letchfield T., O’Farrell-Pearce S. (2020). Dog-assisted interventions and outcomes for older adults in residential long-term care facilities: A systematic review and meta-analysis. Int. J. Older People Nurs..

[51] Klimova B., Toman J., Kuca K. (2019). Effectiveness of the dog therapy for patients with dementia-a systematic review. BMC Psychiatry.

[52] Lundqvist M., Carlsson P., Sjödahl R., Theodorsson E., Levin L.Å. (2017). Patient benefit of dog-assisted interventions in health care: A systematic review. BMC Complement. Altern. Med..

[53] Maber-Aleksandrowicz S., Avent C., Hassiotis A. (2016). A systematic review of animal-assisted therapy on psychosocial outcomes in people with intellectual disability. Res. Dev. Disabil..

[54] Jackson S., Hart L., Brown J., Volkmar F. (2018). Brief report: Self-reported academic, social, and mental health experiences of post-secondary students with autism spectrum disorder. J. Autism Dev. Disord..

[55] Stewart T.M., Martin K., Fazi M., Oldridge J., Piper A., Rhodes S.M. (2022). A systematic review of the rates of depression in autistic children and adolescents without intellectual disability. Psychol. Psychother. Theory Res. Pract..

[56] Flujas-Contreras J.M., Chávez-Askins M., Gómez I. (2023). Efectividad de las intervenciones psicológicas en Trastorno del Espectro Autista: Una revisión sistemática de meta-análisis y revisiones sistemáticas. Rev. Psicol. Clínica Con Niños Adolesc..

